# “Stable-on-the-Table” Biosensors: Hemoglobin-Poly (Acrylic Acid) Nanogel BioElectrodes with High Thermal Stability and Enhanced Electroactivity

**DOI:** 10.3390/s150923868

**Published:** 2015-09-18

**Authors:** Ananta Ghimire, Omkar V. Zore, Vindya K. Thilakarathne, Victoria A. Briand, Patrick J. Lenehan, Yu Lei, Rajeswari M. Kasi, Challa V. Kumar

**Affiliations:** 1 Department of Chemistry, University of Connecticut, Storrs, CT 06269, USA; E-Mails: ananta.ghimire@uconn.edu (A.G.); omkar.zore@uconn.edu (O.V.Z.); vindyakalani@gmail.com (V.K.T.); victoria.briand@gmail.com (V.A.B.); 2 Institute of Materials Science, U-3136, University of Connecticut, Storrs, CT 06269, USA; 3 Department of Molecular and Cell Biology, University of Connecticut Storrs, Storrs, CT 06269, USA; E-Mail: patrick.lenehan@uconn.edu; 4 Department of Chemical and Biomolecular Engineering, University of Connecticut, Storrs, CT 06269, USA; E-Mail: ylei@engr.uconn.edu

**Keywords:** electrochemistry, biocatalysis, hemoglobin, high temperature catalysis, steam sterilization, polyacrylic acid

## Abstract

In our efforts toward producing environmentally responsible but highly stable bioelectrodes with high electroactivities, we report here a simple, inexpensive, autoclavable high sensitivity biosensor based on enzyme-polymer nanogels. Met-hemoglobin (Hb) is stabilized by wrapping it in high molecular weight poly(acrylic acid) (PAA, *M*_W_ 450k), and the resulting nanogels abbreviated as Hb-PAA-450k, withstood exposure to high temperatures for extended periods under steam sterilization conditions (122 °C, 10 min, 17–20 psi) without loss of Hb structure or its peroxidase-like activities. The bioelectrodes prepared by coating Hb-PAA-450k nanogels on glassy carbon showed well-defined quasi-reversible redox peaks at −0.279 and −0.334 V in cyclic voltammetry (CV) and retained >95% electroactivity after storing for 14 days at room temperature. Similarly, the bioelectrode showed ~90% retention in electrochemical properties after autoclaving under steam sterilization conditions. The ultra stable bioelectrode was used to detect hydrogen peroxide and demonstrated an excellent detection limit of 0.5 μM, the best among the Hb-based electrochemical biosensors. This is the first electrochemical demonstration of steam-sterilizable, storable, modular bioelectrode that undergoes reversible-thermal denaturation and retains electroactivity for protein based electrochemical applications.

## 1. Introduction

Redox enzyme based electrodes have attracted increasing interest because of their applications in sensing, biofuel cells and bioelectronic devices [1]. Among many redox proteins, met-hemoglobin (Hb) is considered as a model to study direct electrochemistry-based biosensors, because of its catalytic activity and commercial availability. Although hemoglobin comprises of four iron containing heme groups, its electron transfer activity is hindered due to the extended three dimensional protein envelope around the heme, which buries the electroactive centers away from the electrode surfaces [2]. As a result, only a weak redox current appears with Hb-based electrodes on application of large over voltages. The electron transfer rate may be enhanced by the addition of promoters and mediators such as surfactants [3], polymers [4] and specific nanomaterials [5]. Using different techniques, Hb has been embedded in these films, which led to enhanced electron transfer rates, as well as better adhesion to the electrode surface. This facilitated the use of Hb-based electrodes for biosensing in food, pharmaceutical, clinical and environmental issues. Despite all this progress, a major concern and unmet challenge is the poor stability of Hb and Hb encapsulated derivatives under non-physiological environments, such as high temperatures, extreme pHs, and electrode surface [6].

Improved thermal stabilities of enzyme electrodes will directly enhance the shelf life for storage at ambient temperatures and facilitate biocatalysis and electrocatalysis at higher temperatures. Particularly, steam-sterilizable bioelectrodes will be useful for biomedical applications. Therefore, there is an urgent need to develop methods to stabilize Hb, as an example, and characterize these new materials for use as bioelectrodes. 

A variety of approaches were developed to enhance protein thermal stability, such as (i) intercalation in layered materials [7,8,9,10,11,12,13,14] and (ii) introducing multiple covalent attachment points to a polymer which may lead to restricted unfolding and conformational stabilization [15,16]. Protein properties are enhanced by forming hydrophilic matrix around the protein [17] via the construction of enzyme polymer conjugates [18,19,20,21,22,23].

We are interested in enhancing storage, shelf-life and thermal stabilities of enzymes and proteins by covalent conjugation with water soluble, flexible polymers, such as poly(acrylic acid) (PAA) [24]. The synthesis and characterization of Hb-PAA nanoparticles using low molecular weight PAA (*M*_W_ 8k) was reported earlier and these withstood steam sterilized conditions without significant loss of structure or biological activities. However, the particle nature of Hb-PAA-8k nanoparticle platform is not favorable for electrochemical application because Hb is encapsulated in the polymer nanoparticles, potentially hindering the accessibility of Hb active center to the electrode surface. Additionally, the nanoparticles limit the number of Hb molecules that could be brought close to the electrode surface and some Hb molecules may be far from the electrode surface. Conversely, protein-polymer nanogels could be more amenable to enhance electrical contact between the redox active sites and the electrode surfaces. However, when low molecular weight PAA was used to form Hb-PAA nanogels, Hb was not protected against steam sterilization conditions (122 °C, 17–20 psi, 10 min) [24].

To amend the above-mentioned issues, we currently report the use of high molecular weight PAA (*M*_W_ 450k) to cross-link Hb molecules and form unique Hb-nanogels such that their thermal stability as well electroactivity at the electrode surface may be improved. This can be due to increased rigidity to the local environment surrounding Hb by the higher molecular weight polymer. The mole ratio of Hb:PAA was optimized and its molecular weight increased such that low overlap concentrations of the high molecular weight PAA would favor nanogel formation instead of nanoparticles. In addition, the mole ratio of the crosslinking reagent (carbodiimide) to the number of COOH groups of PAA that are available for linking to the protein has been optimized systematically to produce water-soluble, highly active and thermally stable nanogels. Furthermore, PAA matrix around Hb was further strengthened by cross-linking carboxylic acid groups of PAA with tetraethylenepentamine (TEPA) or polyenthyleneimine (PEI) using the carbodiimide chemistry.

**Scheme 1:**
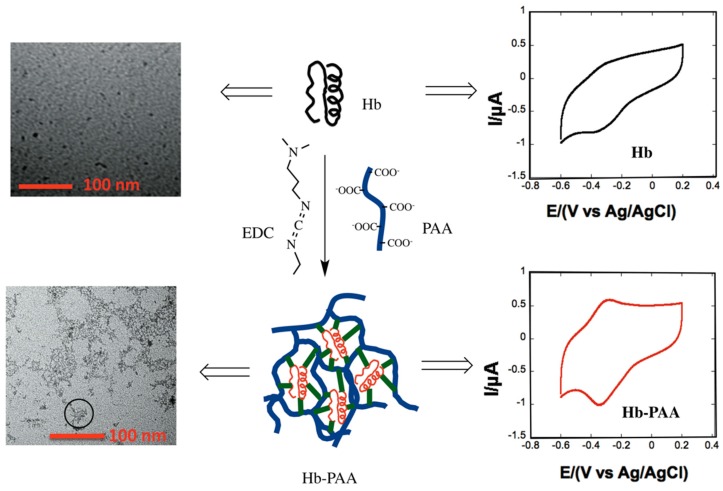
Hemoglobin binding to PAA (**Middle**) and their corresponding cyclic voltammograms (**Right**) and TEM’s (**Left**). TEM image of Hb-PAA shows the formation of nanogels (also shown in circle).

In this manuscript, by optimizing the *M*_W_ of PAA, reactant mole ratios and the crosslinking conditions, we obtained novel Hb-PAA nanogels that are stable at high temperatures and also show excellent electrochemical behavior over extended periods of time, which, to the best of our knowledge, has not been realized before. We also show that Hb, by itself, has poor adhesion to the electrode surface and poor electron transfer rates (Scheme 1, top) while the nanogels are superior. Additionally, Hb in these nanogels undergoes reversible thermal denaturation, while Hb by itself does not (Scheme 1).

More importantly, we exploit this general approach of random conjugation of Hb to PAA to build, for the first time, excellent bioelectrodes, which not only show better adhesion to the electrode surface and enhanced electron transfer rates but also better function at elevated temperatures and these are sterilizable in an ordinary autoclave without significant loss of electroactivity.

## 2. Experimental Section

Poly(acrylic acid ) (*M*_W_ 450k, *M*_W_ 8k), polyethyleneimine (PEI, 8k), tetraethylene pentamine (TEPA), 1-ethyl-3(3-dimethylaminopropyl) carbodiimide (EDC), *o*-methoxyphenol, hydrogen peroxide (H_2_O_2_) were purchased from Sigma-Aldrich (St. Louis, MO, USA). Bovine hemoglobin was obtained from MP Biomedicals (Solon, OH, USA).

### 2.1. Synthesis of Hb-PAA Nanogel Conjugates

PAA stock solution (2 wt % in water) of pH 7 was prepared by dissolving appropriate amount of PAA in DI water and pH was adjusted by adding concentrated NaOH. Hb stock solution (26 mg/mL) was prepared by slowly dissolving the enzyme in 10 mM phosphate buffer at pH 7. According to the amount of PAA and ratio of PAA:EDC (Supplementary Materials Table S1), EDC was mixed with PAA and stirred for 15 min to activate the PAA. Hb in phosphate buffer was then added to EDC activated PAA solution. The reaction was stirred gently for four h. The reaction mixture was dialyzed three times using 25k dialysis membranes against 10 mM phosphate buffer pH 7. Dialyzed conjugates were used for further experiments. The samples were referred to as Hb-PAA-450k(1:0.8:1.5) and Hb-PAA-450k(1:0.3:1.5) based on the molar ratio of Hb:PAA:EDC. Therefore, Hb-PAA-450k(1:0.8:1.5) has Hb:PAA ratio as 1.3:1 and Hb-PAA-450k(1:0.3:1.5) has 3.4:1.

### 2.2. PAA-Hb Nanogel Conjugates Cross-Linking with Polyamines

PEI, *M*_W_ 8k and TEPA were used to cross link selected Hb-PAA conjugates, for example, Hb-PAA-450k(1:0.8:1.5). Stock solutions of polyamines were prepared in DI water and the pH was adjusted to 7 using concentrated HCl. Only 0.2% of COOH groups were modified using TEPA (denoted as Hb-PAA-450k(1:0.8:1.5)-TEPA) and 0.7% of COOH were modified with PEI (denoted as Hb-PAA-450k(1:0.8:1.5)-PEI). Before adding polyamines to the Hb-PAA conjugate, EDC was added to the conjugate (EDC:COOH ratio is 2:1) and stirred 15 min. After addition of TEPA the reaction mixture was stirred another 4 h and dialyzed using 25k dialysis membranes with 10 mM phosphate buffer pH 7 three times.

### 2.3. Circular Dichroism Studies

Secondary and tertiary structure retention of Hb after chemical modification in the presence and absence of PAA was determined using circular dichroism studies. Far UV and Soret CD spectra of Hb, and those in the presence of PAA were recorded using Jasco 710 spectropolarimeter. All samples were in PBS pH 6.4 buffer and the buffer spectrum was subtracted during processing. Step resolution was kept at 0.2 nm/data point and bandwidth and sensitivity were 1 nm and 20 millidegrees, respectively. When collecting far UV CD spectra each sample was scanned from 200 nm to 260 nm and scan speed was maintained at 50 nm/min. Average of four accumulations were recorded using 0.05 cm path length cuvettes. Same samples were used to collect Soret CD spectra and each sample was scanned from 350 to 450 nm and the scan speed was 50 nm/min. Path length used was 1.0 cm and eight accumulations were averaged to get each spectrum.

### 2.4. Catalytic Activity Studies

The catalytic activities of the Hb-PAA nanogel conjugates were evaluated in term of the peroxidase activity of Hb by following reported methods [24] The substrate, O-methoxyphenol (2.5 mM) and the oxidant, H_2_O_2_ (1 mM) were added to the solution containing 1 µM Hb in phosphate buffer pH 7.4.

### 2.5. Heated and Cooled

Hb, Hb-PAA-450k(1:0.8:1.5), Hb-PAA-450k(1:0.3:1.5), Hb-PAA-450k(1:0.8:1.5)-TEPA and Hb-PAA-450k(1:0.8:1.5)-PEI samples were heated at 80 °C for 30 min in an oil bath followed by cooling for 1 h and 24 h at room temperature. Activity studies were performed using molecular devices; flex station 3, plate reader (Sunnyvale, CA, USA) at room temperature after cooling the samples.

### 2.6. Steam Sterilization

Hb, Hb-PAA-450k(1:0.8:1.5), Hb-PAA-450k(1:0.3:1.5), Hb-PAA-450k(1:0.8:1.5)-PEI and Hb-PAA-450k(1:0.8:1.5)-TEPA were steam-sterilized using Amsco Century Scientific (Model SI-120, Steris, Mentor, OH, USA). All liquid samples were steam sterilized for 10 min at 122 °C (17–20 psi). Activity studies were performed after samples were cooled for 1 h and 24 h at room temperature, using molecular devices; flex station 3, plate reader (Sunnyvale, CA, USA).

### 2.7. Calculating K_M_ and V_max_ Values

*K*_M_ and *V*_max_ values of Hb and Hb-PAA conjugates were determined by performing peroxidase activity at different *o*-methoxyphenol concentrations (0.5 mM to 4 mM) using an HP 8453 diode array spectrophotometer (Agilent Technologies, Palo Alto, CA, USA). Lineweaver-Burk plots were constructed by plotting inverse initial rate *vs.* inverse *o*-methoxyphenol concentrations. Michaelis-Menton parameter (*K*_M_) and the maximum velocity (*V*_max_) were extracted from the plot. The concentration of the Hb part in all cases was kept at 1 µM and the H_2_O_2_ concentration was 1 mM.

### 2.8. Dynamic Light Scattering (DLS)

Hydrodynamic radius of Hb and Hb-PAA conjugates were measured using CoolBatch+ dynamic light scattering apparatus, where a Precision detector (Varian Inc. Palo Alto, CA, USA) using a 0.5 × 0.5 cm^2^ cuvette and 658 nm excitation laser source at 90 °C geometry. Samples (0.5 nM Hb or 0.5 nM Hb-PAA in phosphate buffer pH 7.4) were filtered with 0.2-micron filter (PVDF, 13 mm, Fisher Scientific) prior to the measurements. All samples were equilibrated for 300 s at 26 °C and 5 repetitions with 60 accumulations were done at the same temperature. Precision Elucidate Version 1.1.0.9 was used to run the experiment and Deconvolve Version 5.5 was used to process the data.

### 2.9. Elemental Analysis

To confirm the covalent conjugation of PAA (*M*_W_ 450k) to Hb, elemental analysis was carried out with conjugates samples and compared the results with unmodified Hb. All samples after covalent conjugation were dialyzed in 25k *M*_W_ dialysis tubing against deionized water to remove any unreacted EDC. After that water from the sample was removed by freeze-drying using FreeZone 6 Liter Console Freeze Dry System from LABCONCO (Kansas City, MO, USA) (catalog no. 7753024). Freeze-drying was carried out for three days under −25 °C temperature and 0.024 T pressure using the above instrument. After that samples were sent to Galbraith Laboratories Inc. for elemental analysis

### 2.10. Electrochemistry

Cyclic Voltammetry and amperometric experiments were carried out using a Model CHI 601C electrochemical workstation (CH Instruments, Austin, TX, USA). A conventional three-electrode system was used with glassy carbon (GC) as working electrode, Ag/AgCl as reference electrode and platinum as counter electrode. Before electrochemical measurement, the GC electrodes were polished with alumina powder (1 µm and 50 nm respectively). Then the electrode is rinsed carefully with deionized water followed by sonication. After drying, 10 μL of 5 μM Hb-PAA nanogel conjugates was drop casted onto the electrode surface and dried in vacuum for five h. EC experiments were carried out in PBS (0.1 M, pH 7.4). Prior to experiment, all the solutions were purged with nitrogen for 30 min and the nitrogen environment is maintained through the experiment. The charge Q under the reduction peak is calculated by integrating the area under the peak using CHI software.

## 3. Results and Discussion

### 3.1. Conjugate Synthesis

The nanogel conjugates were synthesized by crosslinking Hb and PAA450k using EDC with 1:x mole ratios of Hb to PAA45k, and y:1 mole ratios of EDC to COOH groups in PAA. The conjugates are named as Hb-PAA-450k(1:x:y). We test the hypothesis that the thermal stability and resistance to steam sterilization conditions (122 °C, 40 min, 17–20 psi) will improve by conjugation with the polymer in nanogels by systematically increasing the amount of PAA covalently attached to the protein. Mole ratio of Hb:PAA was altered from 1:0.3 to 1:0.8 such that increased PAA mole ratio augmented the average number of polymer chains attached per protein molecule. This resulted in a thicker polymer matrix around the protein, which could allow for improved stability by inhibiting protein denaturation due to the reduced entropy effect. The polar, water-soluble, ionic polymer shell prevents protein aggregation and it could enhance the reversibility of protein denaturation.

Most mammalian hemoglobins have roughly 44 lysine residues [24] per molecule and most of these amines could be attached to the carboxyl groups of PAA chains using EDC chemistry. The mole ratio of EDC:COOH was increased from 0.13 to 1.5, by more than an order of magnitude to evaluate the influence of the degree of crosslinking on the chemical, physical and biological properties of the Hb-PAA conjugate. Our hypothesis is that strengthening of the polymer shell around the protein would increase protein stability and also improve the extent of reversibility of denaturation. Using EDC chemistry lysine-NH_2_ groups of Hb are conjugated with carboxyl groups of PAA (*M*_W_ 450k). Again, the carboxyl groups of PAA are activated using EDC and the polymer chains are further crosslinked using polyamines TEPA and PEI. Thus, the conjugate was rationally designed and engineered to have the best thermal stability while retaining its enzyme-like activities, as well as its secondary structure, as presented below.

Increase in PAA wt % as well as the mole ratio of EDC:COOH led to rapid heterogeneous gelation (Supplementary Materials Table S1). The samples were labeled based on visible observation of the sample viscosity as L = liquid, TL = thickened liquid, G = homogeneous gel, HG = heterogeneous gel, as in case of Hb-PAA-450k(1:3:0.5) increased molar ratio of PAA resulted in the formation of thickened gel. From this library of Hb-PAA conjugates (Supplementary Materials Tables S1 and S2) two conjugates Hb-PAA-450k(1:0.8:1.5) and Hb-PAA-450k(1:0.3:1.5) of low viscosity were selected for further studies to evaluate their activity, structure and electrochemistry. Hb-PAA-450k(1:0.3:1.5) has nearly three-times excess EDC per COOH of PAA when compared to previously reported Hb-PAA conjugate [24]. Therefore, comparison of these three samples revealed the influence of increased conjugation and increased PAA content of the conjugates. 

Effect of further rigidification of the local environment was evaluated by cross linking Hb-PAA-450k(1:0.8:1.5) with two different polyamines, TEPA and PEI using EDC chemistry (Supplementary Materials Table S5). The influence of polyamine crosslinking was evaluated in steam sterilization studies.

### 3.2. Agarose Gel Electrophoresis

**Figure 1:**
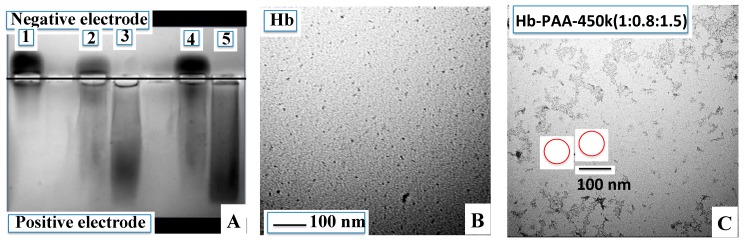
Covalent conjugation of Hb with PAA shown by agarose gel and TEM’s. (**A**) Agarose gel of Hb and Hb-PAA conjugates (40 mM Tris acetate pH 6.5). Lane 1, Hb; lane 2, Hb/PAA-450k(1:0.8:1.5) physical mixture; lane 3, Hb-PAA-450k(1:0.8:1.5); lane 4, Hb/PAA-450k(1:0.3:1.5) physical mixture, and lane 5, Hb-PAA-450k(1:0.3:1.5). Hb migrated towards negative electrode (lane 1) and Hb-PAA migrated (lanes 3 and 5) towards the positive electrode due to the negatively charged carboxyl groups of PAA conjugated to Hb; (**B**) TEM image of Hb (**C**) TEM image of Hb-PAA-450k(1:0.8:1.5) showing nanogels (also shown in circle). All TEM’s are after staining with uranyl acetate.

Agarose gel electrophoresis confirmed the covalent attachment of Hb to PAA (Figure 1A). The gel was run in 40 mM Tris acetate at pH 6.5. At this pH, Hb is slightly positively charged and migrated toward the negative electrode, while Hb-PAA samples migrated toward the positive electrode due to the excess negative charge on PAA. Hb (lane 1) migrated towards the negative electrode and lanes 2 and 4, contained the physical mixtures of Hb and PAA with compositions same as those of Hb-PAA-450k(1:0.8:1.5) and Hb-PAA-450k(1:0.3:1.5). The physical mixtures indicated unbound Hb and mere presence of PAA did not alter Hb migration. In sharp contrast to these lanes, the conjugates Hb-PAA-450k(1:0.8:1.5) and Hb-PAA-450k(1:0.3:1.5) (lanes 3 and 5, respectively) indicated movement in the opposite direction and indicated essentially complete conjugation of Hb to PAA, and there has been no detectable amounts of unbound Hb in these lanes.

### 3.3. Elemental Analysis

Conjugates showed decrease in nitrogen content and increase of carbon content when compared to the corresponding values of Hb. The C:N ratio for Hb is 3.35 while those of Hb-PAA-450k(1:0.3:1.5), Hb-PAA-450k(1:0.8:1.5), Hb-PAA-450k(1:0.8:1.5)-TEPA and Hb-PAA-450k(1:0.8:1.5)-PEI were 5.76, 8.26, 7.98 and 5.64, respectively. The increased C:N ratio proves that the carbon-rich PAA has been conjugated to Hb. Hb-PAA-450k(1:0.8:1.5) showed higher C:N ratio than Hb-PAA-450k(1:0.3:1.5), since mole ratio of Hb:PAA used with Hb-PAA-450k(1:0.8:1.5) was 1:0.8 while that of Hb-PAA-450k(1:0.3:1.5) has been only 1:0.3. In case of cross-linked samples, Hb-PAA-450k(1:0.8:1.5)-PEI showed lesser C:N ratio when compared to Hb-PAA-450k(1:0.8:1.5)-TEPA, since PEI is a polyimine with higher nitrogen content when compared to TEPA. These data strongly support conjugate synthesis and further crosslinking.

### 3.4. TEM and DLS

Morphology of Hb-PAA conjugates were determined using TEM (Experimental in Supplementary Information). Figure 1B shows the TEM image of the Hb in which discrete particles were noted. The TEM of Hb-PAA-450k(1:0.8:1.5) and Hb-PAA-450k(1:0.3:1.5) (Figure 1C, Supplementary Materials Figure S2C) samples clearly show nanogel structures that resulted from cross linking between Hb and PAA. TEM images are consistent with nanogel structure formation with 450 k molecular weight PAA as reported earlier [24]. Extensive network structure was observed with Hb-PAA-450k(1:0.8:1.5)-TEPA and Hb-PAA-450k(1:0.8:1.5)-PEI conjugate samples which arise from excess cross-linking between PAA matrixes (Supplementary Materials Figure S2A,B). As previously reported, lower molecular weight PAA (8k) resulted in nanoparticles [25] but in the current case higher molecular weight PAA (450k) resulted in nanogels. As expected, higher PAA mole ratio (Hb-PAA-450k(1:0.8:1.5)) gave nanogels of larger size compared to that of lower mole ratio (Hb-PAA-450k(1:0.3:1.5)). Similarly, the overall size of the Hb-PAA nanogel decreased upon cross-linking.

Dynamic light scattering (DLS) data (Supplementary Materials Table S3) showed particle sizes of 85 nm for Hb-PAA-450k(1:0.8:1.5) and 63 nm for Hb-PAA-450k(1:0.3:1.5) conjugates. Particle size decreased when cross linked with polyamines, 70 nm for Hb-PAA-450k(1:0.8:1.5)-TEPA and 60 nm for Hb-PAA-450k(1:0.8:1.5)-PEI. The size and formation of soluble nanogels from DLS corroborated well with the TEM and agarose gel electrophoresis data.

### 3.5. Protein Structure Determination

**Figure 2:**
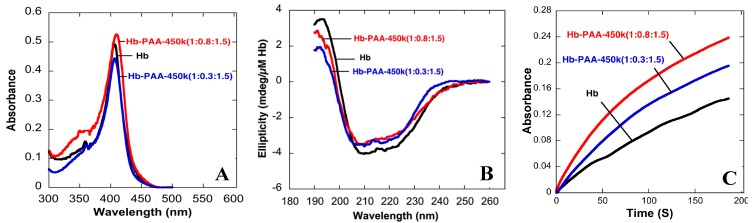
(**A**) Absorbance spectra of Hb, Hb-PAA-450k(1:0.8:1.5) and Hb-PAA-450k(1:0.3:1.5); (**B**) Far UV CD spectra of Hb, Hb-PAA-450k(1:0.8:1.5) and Hb-PAA-450k(1:0.3:1.5); (**C**) Kinetic traces of Hb,. Hb, Hb-PAA-450k(1:0.8:1.5) and Hb-PAA-450k(1:0.3:1.5). All data collected in 10 mM phosphate buffer pH 7.4.

The Soret absorbance bands of Hb-PAA(1:0.8:1.5) and Hb-PAA(1:0.3:1.5) matched well with that of Hb (Figure 2A). The small shifts in the peak positions are negligible, and similar shift of the Soret band was observed upon PEGylation or methylation of Hb [26]. If heme were to be removed from its binding pocket during our conjugation reaction, the Soret peak would have shifted blue to almost ~385 nm, which did not happen with our Hb-PAA samples.

The protein secondary structure was assessed by circular dichroism (CD) spectra (Figure 2B) and the CD spectra of Hb-PAA-450k(1:0.8:1.5), Hb-PAA-450k(1:0.8:1.5)-TEPA and Hb-PAA-450k(1:0.8:1.5)-PEI were compared with that of Hb. All spectra were corrected for Hb concentration, path length and baseline (Supplementary Materials Figure S3). Peak positions and intensities of conjugates in the 190–250 nm, which is characteristic of the protein secondary structure, were consistent with the peak positions of unmodified Hb, and thus indicating significant retention of protein secondary structure for the conjugates. The far UV CD spectrum of Hb (black line) has a peak maximum at 195 nm and two minima at 210 and 220 nm. The same positions were observed for the conjugates; Hb-PAA-450k(1:0.8:1.5) (blue line), Hb-PAA-450k(1:0.8:1.5)-PEI and Hb-PAA-450k(1:0.8:1.5)-TEPA (Supplementary Materials Figure S3) with 70%–90% retention of the peak intensities.

### 3.6. Peroxidase-Like Activity

Prior to doing the electrochemical work, the conjugates were further characterized to ensure the retention of the redox activities of the heme center present in Hb in the conjugates. Even though Hb does not function as an enzyme in biological systems, its peroxidase-like activity is well known and it has been used to assess the biochemical competence of hemoglobin [27].

**Figure 3:**
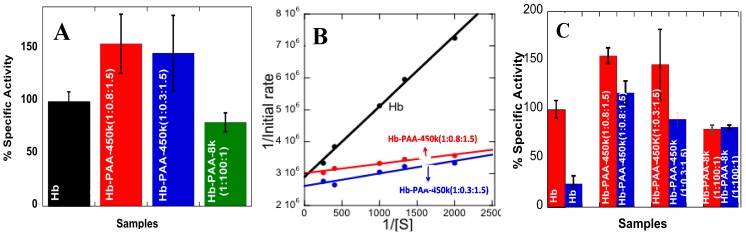
(**A**) Specific activities (compared to Hb, 100%) of Hb-PAA-450k(1:0.8:1.5), Hb-PAA-450k(1:0.3:1.5), and Hb-PAA-8k(1:100:1) at room temperature; (**B**) Lineweaver-Burk plots for peroxidase activities of Hb, Hb-PAA-450k(1:0.8:1.5) and Hb-PAA-450k(1:0.3:1.5) samples; (**C**) Comparison of specific activities of Hb (compared to Hb, 100%), Hb-PAA-450k(1:0.8:1.5), Hb-PAA-450k(1:0.3:1.5), and Hb-PAA-8k(1:100:1) before (red) and after (blue) steam sterilization (cooled for 24 h). Each bar is an average of three trials. All reactions were carried out in the presence of 1 µM protein and 1 mM H_2_O_2_ in phosphate buffer pH 7.4, at room temperature.

Peroxidase-like activities of Hb and its conjugates were monitored by following the oxidation of guaiacol (substrate) by hydrogen peroxide (oxidant). The resulting product absorbs at 470 nm and kinetics were determined from the initial slopes (Figure 2C and Supplementary Materials Figure S1). Specific activities of Hb and Hb-PAA conjugates (Hb-PAA-450k(1:0.8:1.5), Hb-PAA-450k(1:0.3:1.5)), (Hb-PAA-450k(1:0.8:1.5)-PEI and Hb-PAA-450k(1:0.8:1.5)-TEPA) were estimated from the kinetic data (Figure 3A and Supplementary Materials Figure S4). The activities of the conjugates are shown relative to Hb as 100%.

Hb-PAA-450k(1:0.8:1.5) and Hb-PAA-450k(1:0.3:1.5) indicated improved activities (150%) when compared to that of Hb and these are better than that of Hb-PAA-8k (4th bar). The gains were less when the conjugates were cross-linked with polyamines (Supplementary Materials Figure S4). Analysis of the initial rates with increasing substrate concentrations but under similar conditions of buffer, ionic strength and pH by Michaelis-Menton plots and Lineweaver-Burk plots (Figure 3B) to obtain the Michaelis-Menton constant (*K*_M_) and the maximum velocity (*V*_max_) (Table 1) [28], Figure 3B.

**Table 1:** K_M_, *V*_max_, *k*_cat_ and *k*_cat_/*K*_M_ values for Hb, Hb-PAA-450k(1:0.8:1.5) and Hb-PAA-450k(1:0.3:1.5) samples. *V*_max_ values of Hb-PAA conjugates are comparable to Hb and *K*_m_ values decreased considerably for Hb-PAA conjugates in comparison to Hb.

	*K*_M_	*V*_max_	*k*_cat_	*k*_cat_/*K*_M_
Hb	0.76 mM	0.345·µM·s^−1^	0.345·s^−1^	4.54 × 10^2^ M^−1^·s^−1^
Hb-PAA-450k(1:0.8:1.5)	0.10 mM	0.332·µM·s^−1^	0.332·s^−1^	3.32 × 10^3^ M^−1^·s^−1^
Hb-PAA-450k(1:0.3:1.5)	0.15 mM	0.383·µM·s^−1^	0.383·s^−1^	2.55 × 10^3^ M^−1^·s^−1^

The *V*max values of Hb-PAA-450k(1:0.8:1.5) and Hb-PAA-450k(1:0.3:1.5) are comparable to Hb, but the *K*_M_ values decreased seven fold (Hb-PAA-450k(1:0.8:1.5)) when compared to that of Hb. Decreased K_M_ indicates increased substrate affinity to the Hb active site. Hb-PAA-450k(1:0.8:1.5) has smaller *K*_M_ than Hb-PAA-450k(1:0.3:0.5). This would directly increase the overall rate of the catalytic reaction, which is appreciated for practical applications.

### 3.7. Reversibility of Thermal Denaturation

Prior to steam sterilization of the samples, we initially tested the thermal stability of the conjugates by heating the samples beyond the denaturation temperature (80 °C). If the denaturation is reversible, then the heat treated samples would have considerable retention of the Soret band and the catalytic activity. Absorbance spectra of Hb, Hb-PAA-450k(1:0.8:1.5) and Hb-PAA-450k(1:0.3:1.5) samples before heating, at 80 °C and after cooling down to room temperature (Supplementary Materials Figure S5) showed that both Hb-PAA-450k(1:0.8:1.5) and Hb-PAA-450k(1:0.3:1.5) retained ~90% of the Soret band absorbance while Hb lost almost 50% of the Soret band intensity (Supplementary Materials Figure S5B,C). The conjugates underwent reversible thermal denaturation and these conclusions are tested by CD spectra.

The Soret CD spectra of Hb-PAA-450k(1:0.8:1.5), Hb-PAA-450k(1:0.3:1.5) and Hb before and after heating to 80 °C (Supplementary Materials Figure S6). Hb-PAA-450k(1:0.8:1.5) and Hb-PAA-450k(1:0.3:1.5) conjugates retained ~50% of their initial Soret CD while Hb lost almost 70% of the band intensity. Hence, it is evident that at least ~50% of the sample has refolded after thermal denaturation and cooling to room temperature and the extent of recovery depended on cooling time.

The influence of heating and cooling on the peroxidase activities were determined to guage the extent of recovery of activity, not just the secondary structure observed in the CD studies. Hb-PAA-450k(1:0.8:1.5), Hb-PAA-450k(1:0.8:1.5)-TEPA and Hb-PAA-450k(1:0.8:1.5)-PEI heated to 80 °C and cooled only 1 h retained 75%–85% of the initial activities (Supplementary Materials Figure S7) while Hb retained only ~15% of its initial activity. Similarly, Hb-PAA-450k(1:0.3:1.5) retained 50% of its initial activity upon heat treatment followed by cooling for one hour. The activities of Hb-PAA-450k(1:0.8:1.5), Hb-PAA-450k(1:0.3:1.5), Hb-PAA-450k(1:0.8:1.5)-PEI and Hb-PAA-450k(1:0.8:1.5)-TEPA improved after cooling for 24 h to 70%, 65%, 95% and 90%, respectively. The crosslinking with the polyamines improved the extent of activity retention, in support of our hypothesis. These data prompted us to test the thermal stabilities of the conjugates on exposure to steam sterilization conditions as a bench mark for thermal stability.

### 3.8. Stability towards Steam Sterilization

Peroxidase-like activities of conjugates were monitored after the samples were steam sterilized (10 min at 122 °C and 17–20 psi, and cooling for 30 min) and cooled back to room temperature for 24 h (Figure 3C). The samples, Hb-PAA-450k(1:0.8:1.5), Hb-PAA-450k(1:0.8:1.5)-PEI and Hb-PAA-450k(1:0.8:1.5)-TEPA retained almost 80%–90% of their initial activities, while Hb retained only 20% of its initial activity. Thus, (1) re-folding of denatured Hb depends on the extent of crosslinking, cooling time and (2) it is a kinetic phenomenon. Thus, in addition to the use of PAA to prepare and stabilize conjugates, polyamine cross-linking enhanced protein re-folding.

### 3.9. Direct Electrochemistry of Hb-PAA Nanogels

Encouraged by the above improvements in the thermal stabilities of the conjugates and due to their nanogel morphology, we hypothesized that these could serve as good model systems to evaluate their function in bioelectrodes. Direct electron transfer of Hb-derivatives was already demonstrated by other researchers but the stability and activity of these systems have not been systematically investigated. We used Cyclic Voltammetry to study the direct electron transfer of Hb-PAA modified electrodes.

**Figure 4:**
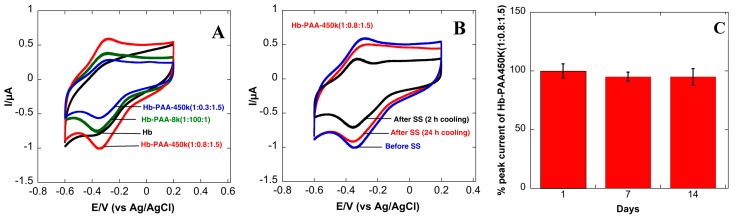
Improved stability: Cyclic voltammograms of Hb-PAA electrodes, (**A**) in pH 7.4 PBS (0.1 M) before heat treatment; (**B**) Hb-PAA-450k(1:0.8:1.5) electrode after steam sterilization (SS) with 2 h cooling (black) and 24 h cooling (red) in pH 7.4 PBS (0.1 M); (**C**) Stability of Hb-PAA-450k(1:0.8:1.5) electrodes at room temperature over 14 days.

Cyclic voltammograms (CVs) of the Hb-electrodes in 0.1 M phosphate buffered (PB, pH 7.4) are shown in Figure 4A. The CV of the Hb- electrode (Scheme 1, right top panel and black curve in Figure 4A showed very poor redox peaks while the electrode modified with Hb-PAA showed a pair of well-defined quasi-reversible redox peaks (red, green and blue). Thus, PAA not only favorably impacts the catalytic efficiency of Hb, but also provide the suitable microenvironment for electron transfer between Hb and the underlying electrode. An increase in catalytic activity and electron transfer rate was seen when Myoglobin (Mb) is covalently linked with PAA functionalized iron nanoparticles [29,30]. Among different Hb-PAA conjugates, Hb-PAA-450k(1:0.8:1.5) displayed more enhanced and sensitive redox peaks than the others. Based on the excellent peroxidase like activity (Figure 3A) and thermal stabilities (Figure 3C), Hb-PAA-450k(1:0.8:1.5) conjugate is chosen to be the best for further electrochemical studies.

The enhanced response of Hb-PAA-450k(1:0.8:1.5) clearly indicated that the direct electron transfer is greatly promoted by increased polymer concentration and increased covalent conjugation. The polymer dependent electron transfer is further verified by comparing the CVs of Hb-PAA-8k Hb-PAA-450k. Due to the highly water-soluble nature of Hb-PAA nanogels, the CVs were collected and compared in presence and absence of nafion after 30 min in 0.1 M PBS with continuous nitrogen bubbling. In both cases, it displayed the same redox peak intensity (Supplementary Materials Figure S8), which indicates Hb-PAA nanogels are well adsorbed on the electrode surface.

The anodic and cathodic peak of Hb in Hb-PAA (Hb-PAA-450k(1:0.8:1.5)) are located at 0.279 V and 0.334 V with peak to peak separation of 55 mV at the scan rate of 100 mV/s. This peak to peak separation is smaller than 70 mV for Nafion/Hb-MWCNT system in ionic liquid modified carbon paste electrode [31], 56 mV for Hb microbelts [32], 70 mV for Hb immobilized in sodium alginate-MWCNT composite film [33] but is higher than Hb at mesoporous carbon modified GC electrode (53 mV) [34] at the same scan rate. This indicates quasi-reversible electron transfer of Hb in the Hb-PAA conjugate and is consistent with the characteristics redox of the heme proteins (Fe(III)/Fe(II)) [35]. The concentration (mol/cm^2^) of electroactive Hb on the electrode surface was determined by integrating the reduction peak according the equation, *Γ* = *Q*/*nFA*, where *Γ* is the concentration of electroactive Hb, *Q* is charge involved in reaction calculated by integrating the reduction peak using software (5.665 × 10^−7^ C), *n* is number of electrons transferred, A is area of the electrode (7.06 × 10^−2^ cm^2^) and F is Faraday constant (96,485 C/mol). Out of 7.08 × 10^−7^ mol/cm^2^ deposited on electrode surface, the concentration of electroactive Hb was estimated to be 8.31 × 10^−11^ mol/cm^2^ (0.01%), assuming one electron transfer. This is 4.4 times higher than the theoretical monolayer coverage of Hb, 1.89 × 10^−11^ mol/cm^2^ [36], which is higher than 1.6 times for Hb entrapped in agarose hydrogel in RT ionic liquid [36], 4.0 times for Hb immobilized in polyacrylamide-P123 [37] but smaller than most of the system with Hb immobilized in CNTs. CNTs are known to bridge between the layers, thus, making the electron hopping possible to larger distance from electrode surface thus resulting 114 times more electroactive species for Hb immobilized in graphene/CNTs [31], 21 times more electroactive species for Hb immobilized in sodium alginate-MWCNT composite film [33]. Our data, in comparison to published literature, suggests multi layers of Hb-PAA are present on the electrode and enough concentration of electroactive Hb species is present in these layers which allows this material to be electroactive even in the absence of conducting carbon inclusions.

### 3.10. Stability of Hb-PAA-450k(1:0.8:1.5) Modified GC Electrodes

#### 3.10.1. Cyclic Voltammetry of Steam Sterilized Samples

The cyclic voltammetry of Hb-PAA-450k(1:0.8:1.5) was recorded before and after steam-sterilization (SS). Figure 4B shows ~65% retention in peak current within 2 h (black) and ~90% after 24 h (red) of cooling at room temperature. This observation further supports the idea that Hb re-folding in Hb-PAA conjugate is a kinetic phenomenon and substantial re-folding occurs within 24 h. The peak intensity of Hb intercalated in zirconium phosphate decreased beyond 85 °C indicating the denaturation of the protein [38].

#### 3.10.2. Stability with Time at Room Temperature

The extended stability of Hb-PAA modified electrode at room temperature was tested by monitoring the electroactivities of three Hb-PAA (Hb-PAA-450k(1:0.8:1.5)) electrodes maintained at ambient conditions. The peak current decreased by only 5% after two weeks, indicating the high stability of the electrodes (Figure 4C) over this time period at room temperature. Most enzymes are usually unstable and the enzyme-electrodes are generally stored at 4 °C. Increased stability of the Hb-PAA electrodes (95% retention in peak current) is comparable to 96% retention reported using Hb loaded onto ZnO nanoparticle/ionic liquid system [39], 95% retention in graphene/Fe_3_O_4_ nanocomposites [40] at 4 °C, over the same time period. The increased stability observed with Hb-PAA offer opportunities to build better biosensors with enhanced room temperature storage or wide temperature range applications in contrast to denaturation and subsequent loss in the electroactivity of unprotected enzymes.

### 3.11. Hb-PAA-450k(1:0.8:1.5) Modified GC Electrode for H_2_O_2_ Detection

The peroxidase-like activities of Hb-PAA conjugates synthesized using 450k PAA in solutions was better than Hb itself. Now, the electrocatalytic activity of the same was investigated using cyclic voltammetry (CV).

**Figure 5:**
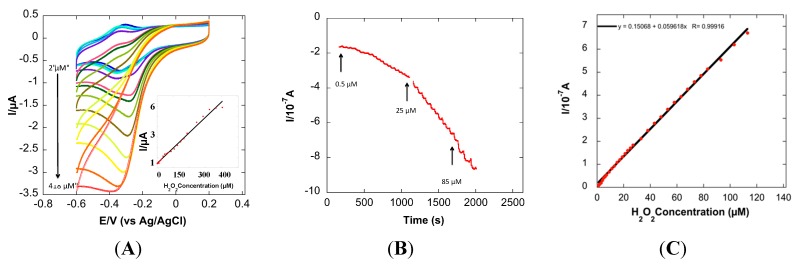
(**A**) Cyclic Volatmmograms of Hb-PAA-450k(1:0.8:1.5) modified GC electrode at different concentration of H_2_O_2_ (Inset: corresponding calibration plot); (**B**) Amperometric response of Hb-PAA modified electrode with successive additions of H_2_O_2_ in PB pH 7.4 at an applied potential of −0.335 V at 25 °C; (**C**) Corresponding calibration plot of amperometric response towards H_2_O_2_.

Figure 5A shows the CVs of Hb-PAA (Hb-PAA-450k(1:0.8:1.5) in absence and presence of different concentration of H_2_O_2_. The peak current increases linearly with H_2_O_2_ concentration and saturates at about 370 μM. The corresponding calibration plot is shown in inset of Figure 5A. Amperometry is more than CV and was used to determine the response of the Hb-PAA modified electrode with different concentration of H_2_O_2_ (Figure 5B) The amperomety showed linear response up to 120 μM H_2_O_2_ (Figure 5C) at an applied potential of −0.335 v with a detection limit of 0.5 μM_._ This detection limit is compared with the recently reported Hb based H_2_O_2_ biosensors, Table 2, and proved that the detection limit of our system is comparable to other Hb/polymer systems albeit with enhanced storage stability and ability to reversibly denature.

**Table 2:** Relevant data from recently reported Hb-based H_2_O_2_ biosensors.

Electrode	Applied Potential	Electrolyte, pH	Detection Limit (μM)	Reference
Hb/PAN-co-PAA	-	0.1 M phosphate, pH 7.0	4.5	[41]
Hb/sodium alginate-MWCNTs	−0.4 V (*vs*. SCE)	0.1 M phosphate, pH 7.0	16.41	[33]
Hb microbelts	−0.377 V (*vs*. Ag/AgCl)	0.1 M phosphate, pH 7.0	0.61	[32]
Hb/MoS_2_	-	0.1 M phosphate, pH 7.0	6.7	[42]
Polystyrene-Block-PAA/Hb	−0.25 V (*vs*. SCE)	0.1 M phosphate, pH 6.5	12	[43]
Hb-PAA-450k	−0.335 V (*vs.* Ag/AgCl)	0.1 M phosphate, pH 7.4	0.5	This study

## 4. Conclusions

We systematically investigated the influence of polymer molecular weight, Hb to polymer mole ratio and the amount of crosslinking on the thermal and electrochemical properties of Hb-PAA nanogels. Nanogels produced with high molecular weight PAA (*M*_W_ 450k) at high Hb:PAA ratio, at high EDC:COOH mole ratios indicated reversible thermal denaturation of conjugated Hb. Interestingly, this Hb-PAA nanogel in the absence of a mediator presented excellent electrochemical response towards H_2_O_2_ with the detection limit of 0.5 μM. Thus, chemical modification of Hb with PAA enhanced direct communication of the Hb redox center with the underlying electrode. These Hb-PAA modified electrodes are stable at room temperature for two weeks with 95% retention in their electrochemical properties, which removes the need for refrigeration of the bioelectrodes. The approach for bioelectrode stabilization is modular, and current approach can be extended to other proteins or enzymes that have appropriate ligation sites on their surfaces.

There are several features of the Hb-PAA nanogels that are likely responsible for the observed retention in biocatalytic and electrocatalytic activity after exposure to denaturing conditions. First, a major factor is the presence of a substantial polymer shroud around the protein and in the nanogel network that effectively prevented the agglomeration and precipitation of the denatured protein. Further crosslinking by amines robust the polymeric shell around the protein and helps in refolding. This polymeric layer also prevented the denaturation of protein at the electrode surface; second, physical confinement of Hb molecules within nanogel networks shield the protein from the external microenvironment and thus enhanced its stability. By increasing the polymer molecular weight, polymer to Hb mole ratio, and with increased EDC:COOH mole ratio, the polymer shroud around Hb is strengthened, thickened or rigidified to enhanced its ability to protect Hb against denaturation and promoted refolding of the denatured protein. This aspect is similar to the effect of networks in shape memory polymers. Finally, crosslinking between the protein and hydrophilic PAA maintained a hydrophilic environment at the electrode surface and protected protein secondary structure and heme retention in the active site. Hydrophilic environment around the protein has been shown to increase electron transfer rates [44] and this could be an important parameter in the current studies.

When all of the above-discussed factors act in concert, and the synergistic result is the increased, resistance of the Hb-electrode to thermal deactivation and promoted efficient electron transfer. Overall, higher resistance to deactivation by heat is a welcome change for applications in high temperature biocatalysis, sterilization and in the production of novel, sterile biomaterials and robust biocatalysts for sensing and fuel cell applications. Since the improvements in the bioelectrode properties are mostly due to the polymer matrix surrounding the protein, our modular approach is likely to be useful in stabilizing bioelectrodes of other enzymes as well, but this needs to be tested in future studies. This hypothesis will be valuable in directing research to enhance bioelectrode stabilities and their electroactivities.

## Supplementary Files

Supplementary File 1
