# Supplementary material for: “Stable-on-the-Table” Biosensors: Hemoglobin-Poly (Acrylic Acid) Nanogel BioElectrodes with High Thermal Stability and Enhanced Electroactivity
Source: Sensors (Basel). 2015 Sep 18;15(9):23868–85. doi: 10.3390/s150923868 (PMC4610568; doi:10.3390/s150923868)
Supplement: Supplementary File 1 [file sensors-15-23868-s001.pdf]

OPEN ACCESS

sensors

ISSN 1424-8220

www.mdpi.com/journal/sensors

Supplementary Information

"Stable-on-the-Table" Biosensors: Hemoglobin-Poly (Acrylic Acid) Nanogel BioElectrodes with High Thermal Stability and Enhanced Electroactivity. *Sensors* 2015, *15*, 23868-23885

Ananta Ghimire [1], Omkar V. Zore [1], Vindya K. Thilakarathne [1], Victoria A. Briand [1], Patrick J. Lenehan [3], Yu Lei [4], Rajeswari M. Kasi [1,2,*] and Challa V. Kumar [1,2,3,*]

[1] Department of Chemistry, University of Connecticut, Storrs, CT 06269, USA;
E-Mails: ananta.ghimire@uconn.edu (A.G.); omkar.zore@uconn.edu (O.V.Z.);
vindyakalani@gmail.com (V.K.T.); victoria.briand@gmail.com (V.A.B.);

[2] Institute of Materials Science, U-3136, University of Connecticut, Storrs, CT 06269, USA;

[3] Department of Molecular and Cell Biology, University of Connecticut Storrs, Storrs, CT 06269, USA; E-Mail: patrick.lenehan@uconn.edu

[4] Department of Chemical and Biomolecular Engineering, University of Connecticut, Storrs, CT 06269, USA; E-Mail: ylei@engr.uconn.edu

* Authors to whom correspondence should be addressed; E-Mails: kasi@uconn.edu (R.M.K.); challa.kumar@uconn.edu (C.V.K.); Tel.: +1-860-486-3213 (C.V.K.); Fax: +1-860-486-2981 (C.V.K.).

Transmission Electron Microscopy (TEM)

Transmission electron microscopy (TEM) was used to determine the morphology of the Hb-PAA conjugates, Hb and crosslinked Hb-PAA conjugates. Tecnai T12 instrument operating at an accelerating voltage of 120 kV was used to obtain TEM images. Hb concentration was 0.026 mg/mL and PAA concentration was 0.007 mg/mL in the samples that were used for TEM experiment. A drop of each sample solution was deposited on a copper grid covered with Fomvar film. Excess solution was blotted away with a piece of filter paper to leave a thin layer of solution on the grid. The sample was left to dry in air, and then stained with Uranyl acetate for 30 min prior to taking images.

Table S1. Synthesis conditions of small library of Hb-PAA conjugates.

Entry	PAA (mg/mL)	EDC/COOH	State [a]
Molar Ratio = 1 Hb/0.3 PAA			
0A	2.6	0.46	L
0F	2.6	1.5	L
1A	10	0.46	TL
2A	20	0.46	G
Molar Ratio = 1 Hb/0.8 PAA			
**Entry**	**PAA (mg/mL)**	**EDC/COOH**	**State [a]**
0G	1.5	1.5	L
0B	2.6	0.13	L
0C	2.6	0.46	L
0D	2.6	1.5	L
1B	10	.13	TL
1C	10	0.46	G
1D	10	1.5	G
2B	20	0.13	TL
2C	20	0.46	HG
2D	20	1.5	HG
Molar Ratio = 1 Hb/3 PAA			
**Entry**	**PAA (mg/mL)**	**EDC/COOH**	**State [a]**
0E	2.6	0.46	L
1E	10	0.46	TL
2E	20	0.46	HG
3E	30	0.46	HG

[a] L = liquid, TL = thickened liquid, G = homogeneous gel, HG = heterogeneous gel.

Table S2. Synthetic conditions used to make Hb-PAA conjugates.

Sample	Hb:PAA (Mole Ratio)	Hb:PAA (Mass Ratio)	EDC:COOH
Hb-PAA-450k(1:0.3:0.5) *	1 Hb:0.3 PAA	1 Hb:2.1 PAA	0.5
Hb-PAA-450k(1:0.3:1.5)	1 Hb:0.3 PAA	1 Hb:2.1 PAA	1.5
Hb-PAA-450k(1:0.8:1.5)	1 Hb:0.8 PAA	1 Hb:5.5 PAA	1.5

* Hb-PAA450k(1:0.3:0.5) synthesis and characterization was reported previously [1].

Table S3. Sizes of conjugates from DLS studies.

Sample	Size
Hb-PAA-450k(1:0.8:1.5)	84.8 nm (94%)
Hb-PAA-450k(1:0.3:1.5)	62.6 nm (92%)
Hb-PAA-450k(1:0.8:1.5)-PEI	59.4 nm (84%)
Hb-PAA-450k(1:0.8:1.5)-TEPA	68.2 nm (82%)

Table S4. Specific activities of Hb, Hb-PAA-450k(1:0.8:1.5) and Hb-PAA-450k(1:0.3:1.5) at increasing guaiacol concentrations.

Guaiacol	Specific Activity x 10^{-3} (M^{-1})		
**Concentration**	**Hb**	**Hb-PAA-450k(1:0.8:1.5)**	**Hb-PAA-450k(1:0.3:1.5)**
0.5 mM	0.525 ±0.023	1.066 ±0.034	1.140 ±0.054
0.75 mM	0.638 ±0.029	1.112 ±0.044	1.179 ±0.106
1 mM	0.743 ±0.037	1.439 ±0.031	1.259 ±0.085
2.5 mM	1.005 ±0.053	1.204 ±0.029	1.434 ±0.075
4 mM	1.118 ±0.057	1.245 ±0.090	1.375 ±0.149

Table S5. Synthetic conditions used to make Hb-PAA-450K(1:0.8:1.5)-amine conjugates. Purified Hb-PAA-450K(1:0.8:1.5) sample were subjected to following synthetic condition to achieve Hb-PAA-450K(1:0.8:1.5)-TEPA and Hb-PAA-450K(1:0.8:1.5)-PEI conjugates. Only 0.2% of total COOH on Hb-PAA-450K(1:0.8:1.5) were modified with the amine.

Sample	NH_2:COOH	EDC:COOH
Hb-PAA-450k(1:0.8:1.5)-TEPA	0.002	0.004
Hb-PAA-450k(1:0.8:1.5)-PEI	0.002	0.004

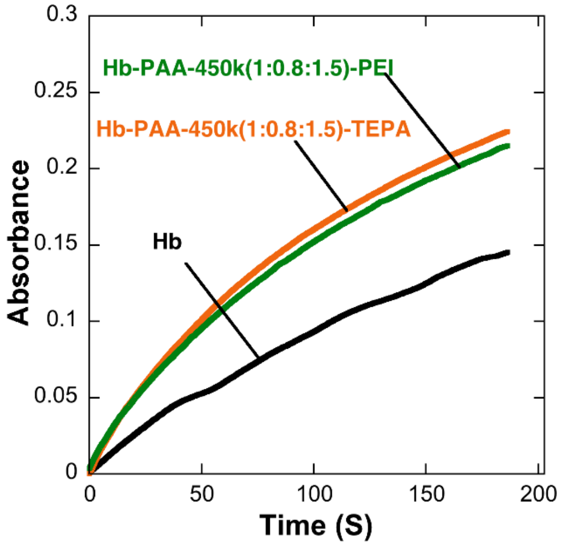

Figure S1. Comparison of kinetic traces of Hb, Hb-PAA-450k(1:0.8:1.5)-TEPA and Hb-PAA-450k(1:0.8:1.5)-PEI. Hb concentration was kept at 1 μM and the H_2O_2 and guaiacol concentrations were 1 and 2.5 mM, respectively. All activity traces were collected at room temperature in phosphate buffer pH 7.4. Each kinetic trace is an average of 4 trials. Initial rates of all samples were extracted from above kinetic traces and corrected for protein concentration to obtain specific activity values.

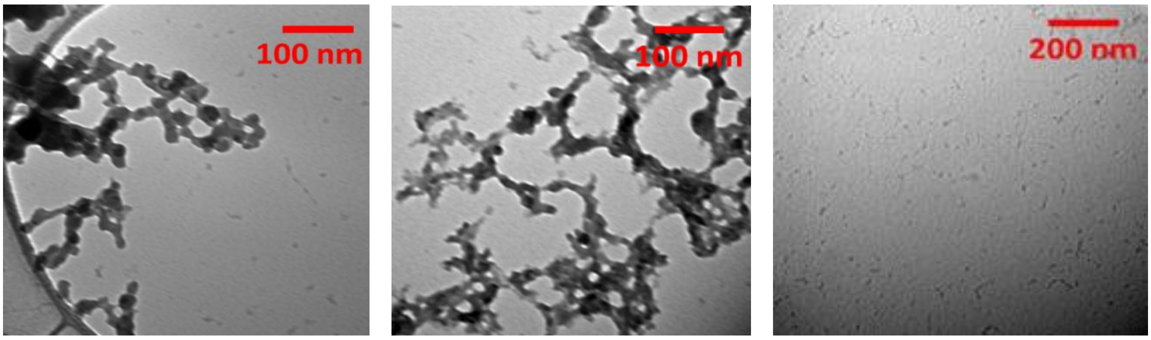

(a) (b) (c)

Figure S2. (**a**) Hb-PAA-450k(1:0.8:1.5)-TEPA; (**b**) Hb-PAA-450k(1:0.8:1.5)-PEI and (**c**) Hb-PAA-450k(1:0.3:1.5) in phosphate buffer pH 7.4, after staining with uranyl acetate. Samples that are crosslinked with polyamines show extensive network structure in TEM micrographs.

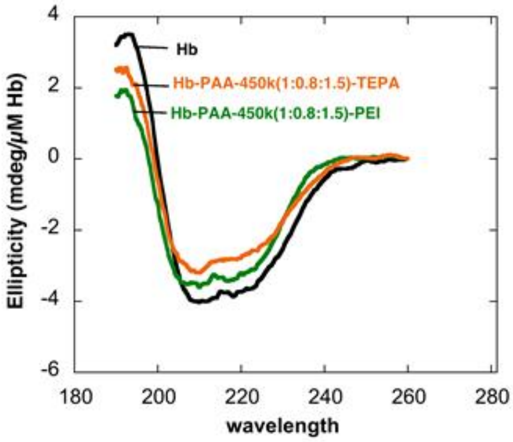

Figure S3. Far UV CD spectra of Hb, Hb-PAA-450k(1:0.8:1.5)-PEI(green) and Hb-PAA-450k(1:0.8:1.5)-TEPA(orange). All the spectra were corrected for Hb concentration and samples were in 10 mM phosphate buffer pH 7.4.

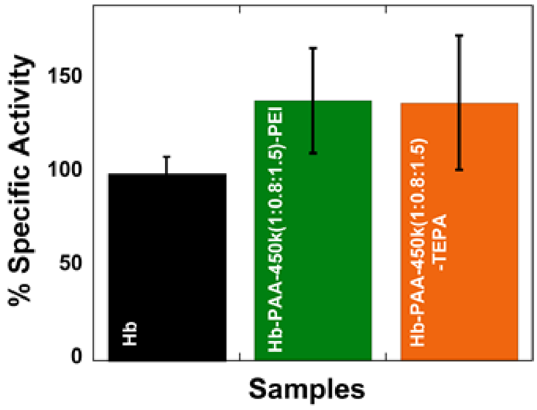

Figure S4. Specific activities (compared to Hb, 100%) of Hb-PAA-450k(1:0.8:1.5)-PEI, Hb-PAA-450k(1:0.8:1.5)-TEPA at room temperature. Hb concentration was 1 μM and the H_2O_2 and guaiacol concentrations were 1 mM and 2.5 mM, respectively. All activity traces were collected at room temperature in phosphate buffer pH 7.4.

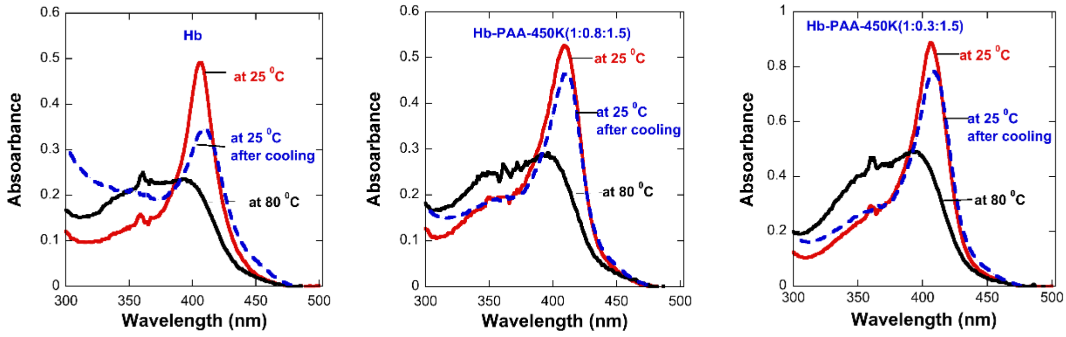

(**a**) (**b**) (**c**)

Figure S5. Absorbance spectra of Hb (**a**), Hb-PAA-450k(1:0.8:1.5) (**b**) and Hb-PAA-450k (1:0.3:1.5) (**c**) at room temperature (red line), heated at 80 ℃ (black line) and cooled for 24 h (blue dash line) at room temperature. All the spectra were corrected for Hb concentration and samples were in 10 mM phosphate buffer pH 7.4.

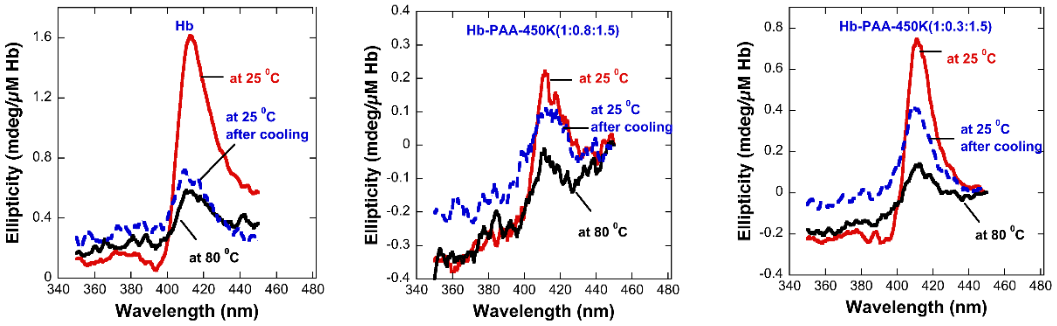

(**a**) (**b**) (**c**)

Figure S6 Soret circular dichroism spectra of Hb (**a**), Hb-PAA-450k(1:0.8:1.5) (**b**) and Hb-PAA-450k(1:0.3:1.5), (**c**) at room temperature (red line), heated at 80 ^{0}C (black line) and cooled for 24 h (blue dashed line) at room temperature. All spectra were normalized with respect to protein concentration and path length (10 mM phosphate buffer pH 7.4).

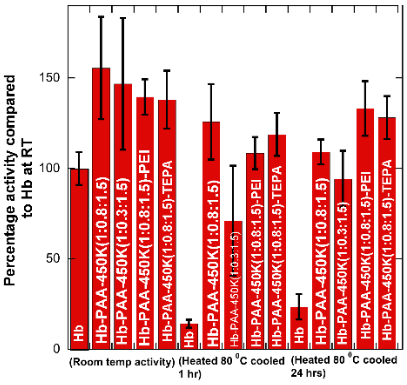

Figure S7. Specific activities (compared to Hb as 100%) of Hb-PAA-450k(1:0.8:1.5), Hb-PAA-450k(1:0.3:1.5), Hb-PAA-450K(1:0.8:1.5)-PEI and Hb-PAA-450k(1:0.8:1.5)-TEPA at room temperature, after heating to 80 ^{0}C and cooling for 1 h, and 24 h. Hb concentration was 1 μM and the H_2O_2 and guaiacol concentrations were 1 and 2.5 mM, respectively. All activity traces were collected at room temperature in phosphate buffer, pH 7.4.

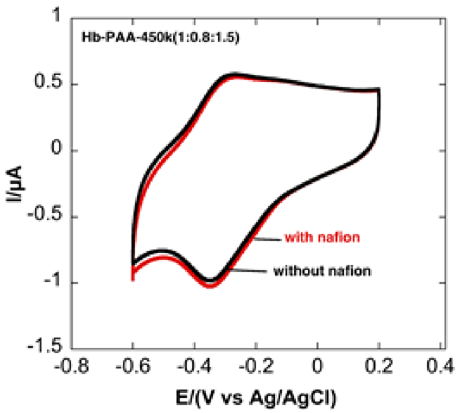

Figure S8. Cyclic voltammograms of Hb-PAA-450k(1:0.8:1.5) with and without nafion immersed in 0.1 M PBS pH 7.4 after 30 min.

References

1. Thilakarathne, V.; Briand, V.A.; Zhou, Y.; Kasi, R.M.; Kumar, C.V. Protein Polymer Conjugates: Improving the Stability of Hemoglobin with Poly(acrylic acid). *Langmuir* **2011**, *27*, 7663–7671.

© 2015 by the authors; licensee MDPI, Basel, Switzerland. This article is an open access article distributed under the terms and conditions of the Creative Commons Attribution license (http://creativecommons.org/licenses/by/4.0/).
